# Cerebrospinal fluid biomarkers for cerebral amyloid angiopathy with non‐hemorrhagic imaging marker

**DOI:** 10.1002/dad2.70431

**Published:** 2026-08-19

**Authors:** Alexander Sekita, Maximilian I. Sprügel, Stefanie Balk, David Haupenthal, Tobias Engelhorn, Manuel Alexander Schmidt, Arnd Doerfler, Timo Jan Oberstein, Juan Manuel Maler, Johannes Kornhuber, Piotr Lewczuk, Veit Rothhammer, Stefan Schwab, Joji B. Kuramatsu, Jochen A. Sembill

**Affiliations:** ^1^ Department of Neurology University Hospital Erlangen and Friedrich‐Alexander‐Universität Erlangen‐Nürnberg Erlangen Germany; ^2^ Department of Neuroradiology University Hospital Erlangen and Friedrich‐Alexander‐Universität Erlangen‐Nürnberg Erlangen Germany; ^3^ Department of Psychiatry and Psychotherapy University Hospital Erlangen and Friedrich‐Alexander‐Universität Erlangen‐Nürnberg Erlangen Germany; ^4^ Department of Neurodegeneration Diagnostics and Department of Biochemical Diagnostics Medical University of Bialystok University Hospital of Bialystok Bialystok Poland

**Keywords:** amyloid, Boston Criteria, cerebral amyloid angiopathy, cerebrospinal fluid biomarkers, non‐hemorrhagic markers

## Abstract

**INTRODUCTION:**

Reduced cerebrospinal fluid (CSF) amyloid beta (Aβ) 40 has been reported in cerebral amyloid angiopathy (CAA) diagnosed by hemorrhagic magnetic resonance imaging (MRI) markers. Whether similar alterations occur when fulfilling Boston Criteria 2.0 solely through non‐hemorrhagic markers remains unclear.

**METHODS:**

We analyzed 358 memory clinic patients undergoing MRI and CSF assessment. CAA was categorized by hemorrhagic markers (CAA 1.5 H) or exclusively non‐hemorrhagic markers (CAA 2.0 NH) per Boston Criteria 1.5 and 2.0, respectively. Primary comparison of CSF–Aβ40 between CAA groups was complemented by secondary (Aβ42, phosphorylated tau [p‐tau181], total tau [t‐tau]) and exploratory analyses including Alzheimer's disease (AD), mild cognitive impairment (MCI), and healthy controls, adjusted for age, sex, MRI characteristics, and assay type.

**RESULTS:**

Sixty‐five patients were reclassified as CAA 2.0 NH. Aβ40 was higher in CAA 2.0 NH than CAA 1.5 H (15,808 vs. 13,792 pg/ml, *p* = 0.033) and did not differ from AD, MCI, or controls. p‐tau181 reached significance after adjustment (*p* = 0.010), likely reflecting AD co‐pathology. However, within CAA‐2.0‐NH, patients with AD CSF profile showed Aβ40 comparable to hemorrhagic CAA.

**DISCUSSION:**

Non‐hemorrhagic imaging markers alone were not associated with characteristic CAA‐related CSF alterations, suggesting biological heterogeneity, limited specificity, or lesion type–dependent variability.

## Highlights

- Non‐hemorrhagic markers identified 65 additional possible cerebral amyloid angiopathy (CAA) cases in the cohort.
- Patients with CAA Boston Criteria 2.0 non‐hemorrhagic markers (CAA 2.0 NH) lacked the distinct cerebrospinal fluid (CSF) biomarker pattern seen in CAA Boston Criteria 1.5 hemorrhagic markers.
- CSF amyloid beta 40 levels were lower in hemorrhagic but not in non‐hemorrhagic CAA.
- CSF biomarkers did not differentiate CAA 2.0 NH from Alzheimer's disease, mild cognitive impairment, or healthy controls.
- Our findings question the specificity of Boston 2.0 criteria non‐hemorrhagic markers in memory clinic populations.

## 1 BACKGROUND

Sporadic cerebral amyloid angiopathy (CAA) is an age‐related small vessel disease characterized by progressive amyloid beta (Aβ) deposition within the walls of small cortical and leptomeningeal blood vessels.^1^ Clinically, CAA is a major cause of lobar intracerebral hemorrhage (ICH) and is associated with cognitive impairment, dementia, and transient focal neurological episodes (TFNE).^1^, ^2^

Definitive diagnosis of CAA remains challenging, as it necessitates histopathological confirmation through brain autopsy.^3^ The modified Boston Criteria provide a non‐invasive in vivo method to diagnose CAA based exclusively on clinical information and magnetic resonance imaging (MRI) markers. Initially, these criteria focused on hemorrhagic MRI findings, which, while specific, likely reflect advanced and irreversible disease stages.^4^, ^5^ In response to this limitation, the updated modified Boston Criteria 2.0, published in 2022, incorporated non‐hemorrhagic MRI markers to enhance diagnostic sensitivity and enable earlier detection of CAA before hemorrhagic complications arise.^3^ This revision also introduced the possibility of diagnosing “possible CAA” based solely on non‐hemorrhagic MRI markers.^3^ However, this also carries the risk of overdiagnosis, given the association of these imaging markers with aging and their prevalence in other conditions, including vascular risk factor–related atherosclerotic disease and Alzheimer's disease (AD).^6^, ^7^

The integration of non‐imaging biomarkers, such as cerebrospinal fluid (CSF) biomarkers, may further refine and expedite the in vivo diagnosis of CAA.^8^ Previous studies reported characteristic CSF biomarker alterations in patients with CAA, particularly reduced Aβ40 levels and modest increases in tau protein.^9^, ^10^ These neurochemical dementia diagnostic (NDD) biomarkers may support differentiation of CAA, as defined by MRI‐based modified Boston Criteria version 1.5, from healthy controls (HCs), patients with mild cognitive impairment (MCI), and patients with AD. However, their diagnostic utility remains uncertain due to overlap with AD pathology, heterogeneity in CAA definitions, variable results across studies with lack of validated thresholds, and the absence of pathological confirmation in most cohorts.^9^, ^10^

The present study primarily aimed to investigate the CSF biomarker profile of patients classified as CAA based solely on non‐hemorrhagic imaging markers in a well‐characterized memory clinic cohort.^9^ CSF biomarker levels, particularly Aβ40 levels, were compared between patients with CAA defined by non‐hemorrhagic versus hemorrhagic markers, as well as with patients with AD, MCI, AD‐MCI, and HCs. Secondary aims were to describe the prevalence of CAA Boston Criteria 2.0 non‐hemorrhagic markers (CAA 2.0 NH) in this cohort and to explore differences in clinical characteristics between groups.

## 2 METHODS

### 2.1 Study design and participants

We retrospectively analyzed 358 patients from the BALANCING (Cerebrospinal Fluid Biomarkers in Cerebral Amyloid Angiopathy) cohort study who were admitted to the Department of Neurology or Department of Psychiatry and Psychotherapy at the University Hospital Erlangen between 2009 and 2018 for diagnostic evaluation of cognitive complaints.^9^ Inclusion required CSF NDD biomarkers and hemosiderin‐sensitive MRI performed within 6 months of lumbar puncture.^9^ Implementing the Boston Criteria 2.0 enabled the diagnosis of possible CAA in additional individuals from previous comparison groups, in the event of present non‐hemorrhagic markers.^3^ Similarly, some patients from the original CAA 1.5 cohort underwent an upgrade from the “possible” to the “probable” category due to implementation of non‐hemorrhagic markers. Diagnosis of AD was made in line with National Institute on Aging and the Alzheimer's Association guidelines.^11^ Cognitively unimpaired individuals without evidence of amyloid‐related disease and without clinical or imaging evidence of cerebrovascular disease were considered HCs. Patients diagnosed with other neurodegenerative diseases were excluded. The University Ethics Committee of Erlangen‐Nuremberg provided approval for the study and granted a central waiver of informed consent.

**RESEARCH IN CONTEXT**

1. **Systematic review**: A literature search via established databases (e.g., PubMed) identified observational studies and meta‐analyses reporting a characteristic cerebrospinal fluid (CSF) biomarker profile in cerebral amyloid angiopathy (CAA), notably decreased amyloid beta 40 in cases diagnosed by hemorrhagic magnetic resonance imaging markers. However, the diagnostic value of CSF biomarkers in patients classified solely by non‐hemorrhagic imaging markers remains underexplored.
2. **Interpretation**: Patients with non‐hemorrhagic CAA markers lack the distinctive CSF profile typically observed in those with hemorrhagic markers. Non‐hemorrhagic markers increased possible CAA diagnosis, but the patients’ biomarker profiles resembled those of patients with Alzheimer's disease, mild cognitive impairment, or controls. This suggests either limited specificity in memory clinic settings, different CAA subtypes or stages, or comorbidities affecting CSF value.
3. **Future directions**: Prospective, multicenter studies with pathological confirmation are needed to assess the diagnostic value of non‐hemorrhagic and CSF markers for CAA in memory clinic cohorts, focusing on their role in differential diagnosis and disease prognosis.

### 2.2 Data acquisition

**FIGURE 1:**
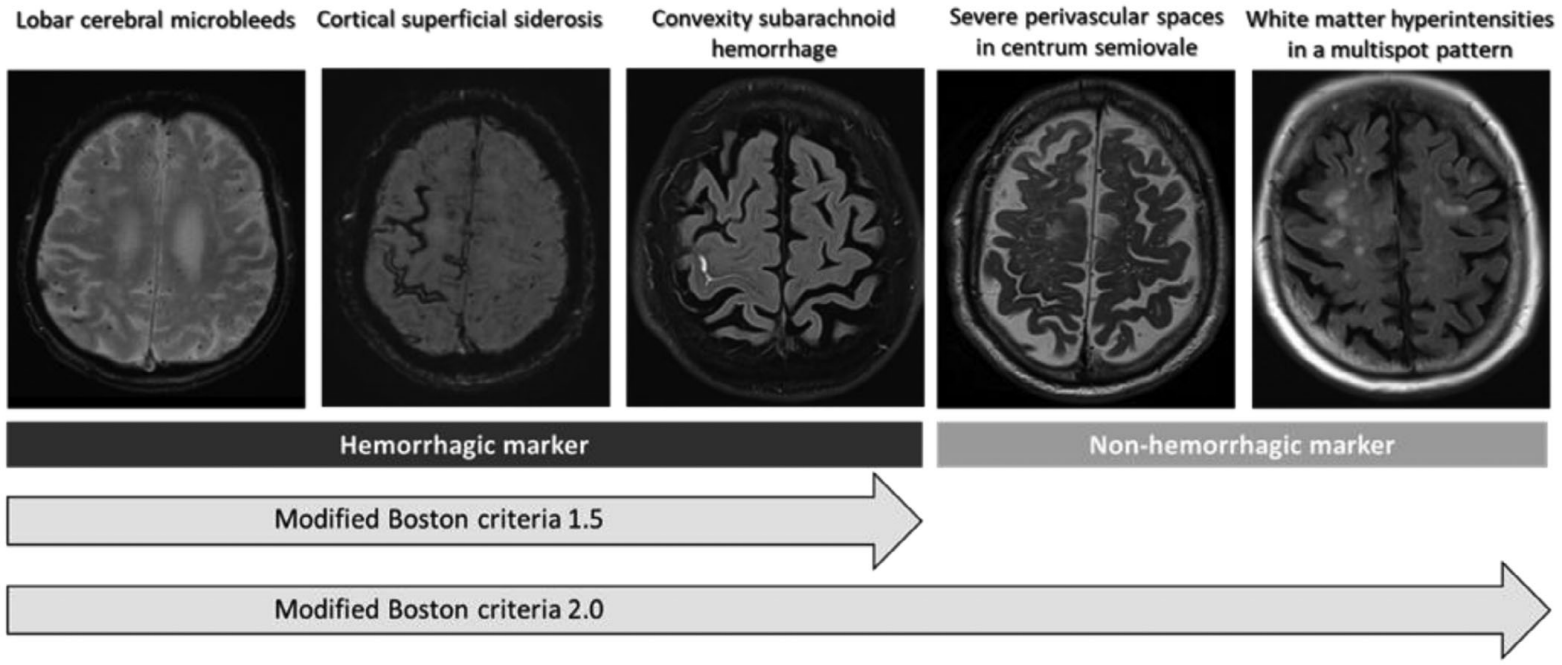
Key brain MRI markers included in the Boston Criteria 2.0 for the diagnosis of cerebral amyloid angiopathy. The figure illustrates hemorrhagic imaging markers, including lobar cerebral microbleeds, cortical superficial siderosis, and convexity subarachnoid hemorrhage, as defined in the modified Boston Criteria 1.5. In the updated Boston Criteria 2.0, non‐hemorrhagic imaging markers are additionally considered, including severe perivascular spaces in the centrum semiovale and multifocal subcortical white matter hyperintensities. Representative MRI sequences used for visualization of the respective imaging markers are as follows: T2*‐weighted GRE and/or SWI for lobar cerebral microbleeds and cortical superficial siderosis; T2*‐weighted GRE and/or SWI or FLAIR imaging for convexity subarachnoid hemorrhage; T2‐weighted imaging for perivascular spaces; and FLAIR imaging for subcortical white matter hyperintensities. FLAIR, fluid‐attenuated inversion recovery; GRE, gradient echo; MRI, magnetic resonance imaging; SWI, susceptibility‐weighted imaging

Baseline characteristics, including demographics, medical history, comorbidities, and clinical symptoms were obtained through chart review and prospective databases, as previously described.^9^ Levels of CSF NDD biomarkers, that is, Aβ40, Aβ42, phosphorylated tau (p‐tau)181, and total tau (t‐tau), were obtained from our prospective laboratory database after analysis in the Laboratory for Clinical Neurochemistry and NDD using commercially available enzyme‐linked immunosorbent assays according to manufacturer protocols.^9^ Specifically, CSF samples were collected by lumbar puncture into polypropylene tubes to minimize adsorption of amyloid peptides and were processed and stored at −80°C until analysis according to standardized laboratory protocols. Aβ42 was measured using assays from IBL International and Fujirebio Europe (formerly Innogenetics); Aβ40 was measured using assays from The Genetics Company and IBL International; t‐tau and p‐tau181 were measured using assays from Fujirebio.^9^ During the study period (2009–2018), assay platforms were changed (May 2010 for Aβ42/40; August 2014 for remaining biomarkers).^9^ All transitions of laboratory protocols and diagnostic‐relevant reference ranges were strictly monitored and validated according to the ISO 15189 Norm.^9^ Biomarker measurements were performed as part of routine clinical diagnostics rather than in batch. Intra‐assay and inter‐assay coefficients of variation were < 10% and < 15%, respectively. Internal laboratory quality control procedures were conducted in accordance with ISO 15189 standards throughout the study period.^9^ To account for potential inter‐assay variability, all statistical analyses were adjusted for assay type. Experienced neuroradiologists, blinded to the clinical data, retrospectively reviewed imaging data acquired on 1.5T or 3.0T MRI scanners. MRI protocols included hemosiderin‐sensitive sequences, that is, T2*‐weighted gradient echo (GRE) or susceptibility‐weighted imaging (SWI), as well as conventional MRI sequences (T1‐, T2‐weighted, and fluid‐attenuated inversion recovery [FLAIR] imaging). Patients were categorized as either possible or probable CAA according to modified Boston Criteria 1.5, encompassing evaluation of patient age, macrohemorrhages, microhemorrhages, and cortical superficial siderosis. Additional categorization according to Boston Criteria 2.0 included assessment of non‐hemorrhagic imaging markers, that is, severe perivascular spaces in the centrum semiovale (> 20 in one hemisphere) and multifocal subcortical white matter hyperintensities (> 10 subcortical FLAIR dots bilaterally), see Figure 1.^3^, ^9^

### 2.3 Outcomes

We analyzed CSF NDD biomarker profiles and patient characteristics. CSF biomarker profiles of patients fulfilling Boston Criteria 2.0 exclusively through non‐hemorrhagic imaging markers (referred to as CAA 2.0 NH) were compared to those of patients fulfilling Boston Criteria 1.5 through hemorrhagic imaging markers (referred to as CAA 1.5 H). The CAA 1.5 H cohort included patients fulfilling either possible (*n* = 38) or probable (*n* = 29) CAA according to modified Boston Criteria 1.5 based on hemorrhagic imaging markers. Patients classified as CAA 2.0 NH did not fulfill hemorrhagic Boston Criteria 1.5 but fulfilled Boston Criteria 2.0 for possible CAA based solely on non‐hemorrhagic imaging markers. Patients with a history of deep ICH or traumatic subarachnoid hemorrhage who did not fulfill CAA 1.5 H criteria but showed non‐hemorrhagic CAA markers remained assigned to their original diagnostic categories, as these hemorrhagic lesions are not considered CAA related. The primary outcome was the comparison of Aβ40 concentrations between these groups. Secondary analyses assessed Aβ42, p‐tau181, and t‐tau levels. Additional exploratory comparisons of CSF biomarker levels were performed with patients with AD, AD‐MCI, MCI, and HCs. These exploratory comparisons were designed to assess group‐level associations in CSF biomarker patterns rather than to establish diagnostic discrimination between individual disease entities. Secondary objectives were to describe the prevalence of CAA 2.0 NH within the cohort and to explore differences in clinical characteristics between groups.

### 2.4 Statistical analyses

Statistical analyses were performed using SPSS (version 28.0, SPSS Inc.) and GraphPad Prism (version 8, GraphPad Software). Statistical tests were two sided and the significance level was α = 0.05. Results are presented as means ± standard deviations (SDs), medians with interquartile ranges (IQRs), or absolute and relative frequencies. Categorical variables were compared using chi‐squared tests, and continuous data were analyzed using Mann–Whitney *U* or Kruskal–Wallis tests for non‐normally distributed variables, as appropriate. CSF biomarker levels were visualized via violin plots. Adjusted linear regression analyses were performed to assess associations between diagnostic groups and CSF biomarker levels while accounting for potential confounders, including age, sex, and NDD biomarker assay characteristics. Because MRI field strength and sequence type may influence the detection sensitivity of hemorrhagic and non‐hemorrhagic imaging markers, separate adjusted regression models additionally included MRI field strength (1.5T vs. 3.0T) and hemosiderin‐sensitive MRI sequence type (GRE vs. SWI) as covariates. To investigate whether CSF biomarker alterations might be driven by concomitant AD pathology rather than CAA itself, CSF biomarker concentrations were compared according to the presence of an AD‐like CSF biomarker profile, defined by decreased CSF Aβ42 and increased p‐tau181 concentrations based on established institutional cutoffs. Comparison of CSF Aβ40 concentrations between CAA 1.5 H and CAA 2.0 NH was defined as the primary analysis. Bonferroni correction for secondary endpoints (Aβ42, p‐tau181, t‐tau; adjusted significance threshold: α = 0.017) was applied consistently across all analyses presented, including the primary comparison between CAA 1.5 H and CAA 2.0 NH, the stratified subgroup analyses, and the exploratory comparisons between CAA 2.0 NH and AD, AD‐MCI, MCI, or HC groups. All subgroup and exploratory analyses beyond the primary comparison are considered hypothesis generating and should be interpreted cautiously, requiring validation in independent cohorts. It should be acknowledged that using CSF Aβ42 and p‐tau181 for both group stratification and outcome comparison introduces potential circularity. Therefore, the focus of the AD‐stratified analysis is particularly on Aβ40, which was not used for AD profile classification and thus remains an independent outcome variable.

## 3 RESULTS

### 3.1 Patient flow and characteristics

Of the 358 patients included in the BALANCING cohort study, 67 patients already fulfilled the CAA 1.5 H criteria. Of these, 38 were diagnosed as possible and 29 as probable CAA based on hemorrhagic imaging markers alone. Application of Boston Criteria 2.0 with additional assessment of non‐hemorrhagic imaging markers resulted in reclassification of 27 patients from possible to probable CAA, leaving 11 patients with possible and 56 patients with probable CAA.

**FIGURE 2:**
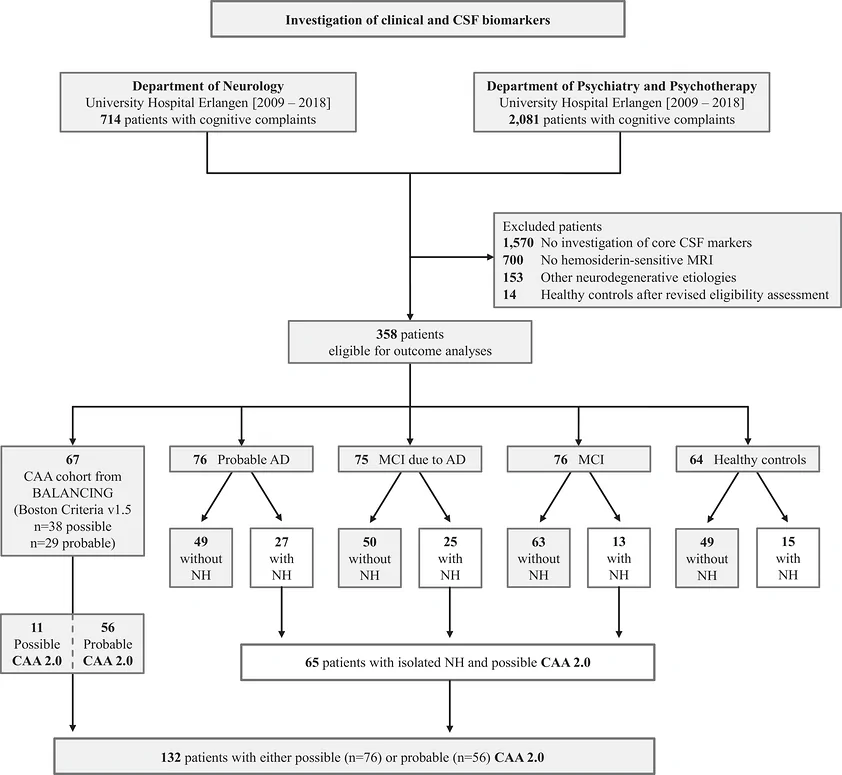
Patient flowchart. Between 2009 and 2018, a total of 2795 patients with cognitive complaints were evaluated at the Departments of Neurology and Psychiatry and Psychotherapy at the University Hospital Erlangen. After exclusion of patients without core CSF biomarker assessments, hemosiderin‐sensitive MRI sequences, or with alternative neurodegenerative diagnoses, 358 patients remained for outcome analyses. Application of the Boston Criteria 2.0 identified 65 additional patients fulfilling possible CAA criteria based solely on non‐hemorrhagic imaging markers. Among patients already fulfilling modified Boston Criteria 1.5 based on hemorrhagic imaging markers, application of Boston Criteria 2.0 additionally resulted in reclassification of several patients from possible to probable CAA due to the presence of non‐hemorrhagic imaging markers. AD, Alzheimer's disease; BALANCING, Cerebrospinal Fluid Biomarkers in Cerebral Amyloid Angiopathy; CAA, cerebral amyloid angiopathy; CSF, cerebrospinal fluid; MCI, mild cognitive impairment; MRI, magnetic resonance imaging; NH, non‐hemorrhagic

Among the remaining 227 patients with cognitive deficits not fulfilling CAA 1.5 H criteria, 65 (29%) were reclassified as CAA 2.0 NH based on the presence of non‐hemorrhagic imaging markers consistent with possible CAA according to Boston Criteria 2.0. This group included 27/76 (36%) patients from the former AD group, 25/75 (33%) patients from the former AD‐MCI group, and 13/76 (17%) patients from the former MCI group. Non‐hemorrhagic imaging markers consisted of 23/67 (34.3%) and 39/65 (60%) of severe perivascular spaces in the centrum semiovale and in 39/67 (58.2%) and 35/65 (54%) of white matter hyperintensities in a multispot pattern in patients with CAA 1.5 H and CAA 2.0 NH, respectively. Application of Boston Criteria 2.0 resulted in 132 patients fulfilling either possible (*n* = 76) or probable (*n* = 56) CAA criteria. The remaining study population consisted of 49 patients with AD, 50 patients with MCI due to AD, 63 patients with MCI, and 64 HCs; see Figure 2. Detailed MRI characteristics are reported in Table S1 in supporting information.

Compared to patients with CAA 1.5 H, patients with CAA 2.0 NH had less frequent arterial hypertension (CAA 2.0 NH: 44 [68%] vs. CAA 1.5 H: 56 [84%], *p* = 0.033), diabetes mellitus (7 [11%] vs. 17 [25%], *p* = 0.030), transient focal neurologic episodes (1 [2%] vs. 8 [12%], *p* = 0.015), and gait disturbances (6 [9%] vs. 16 [24%], *p* = 0.024); see Table 1. Median age was comparable in both groups (73 [67–77] vs. 75 [68–79]) whereas female sex was numerically higher in patients with CAA 2.0 NH (37 [57%] vs. 27 [40%], *p* = 0.060). Comparing patients with CAA 2.0 NH to other etiologies with cognitive impairment showed no relevant imbalances except older age (73 [67–77] vs. 68 [59–75], *p* = 0.002); see Table S2 in supporting information. A detailed intergroup comparison of characteristics between patients with CAA 2.0 NH and patients in their original diagnostic category is shown in Tables S3–S6 in supporting information.

**TABLE 1:** Intergroup comparison of patient characteristics and core cerebrospinal fluid biomarker levels.

				CAA 2.0 NH divided into subgroups		
Characteristics	CAA 1.5 H (*n* = 67)	CAA 2.0 NH^*^ (*n* = 65)	*p* value^†^	MCI‐NH (*n* = 13)	AD‐NH (*n* = 27)	AD‐MCI‐NH (*n* = 25)
Age, years, median (IQR)	75 (68–79)	73 (67–77)	0.18	69 (61–75)	75 (69–77)	73 (69–75)
Sex, female, n (%)	27 (40%)	37 (57%)	0.06	7 (54%)	15 (56%)	15 (60%)
Prior medical history, *n* (%)						
Intracerebral hemorrhage	11 (16%)	0 (0%)	< 0.001	0 (0%)	0 (0%)	0 (0%)
Ischemic stroke	19 (28%)	10 (15%)	0.07	4 (31%)	5 (19%)	1 (4%)
Transient ischemic attack	3 (5%)	2 (3%)	0.67	0 (0%)	2 (7%)	0 (0%)
Subarachnoid hemorrhage	2 (3%)	0 (0%)	0.16	0 (0%)	0 (0%)	0 (0%)
Arterial hypertension	56 (84%)	44 (68%)	0.033	7 (54%)	22 (82%)	15 (60%)
Diabetes mellitus	17 (25%)	7 (11%)	0.030	0 (0%)	3 (11%)	4 (16%)
Prior medication, *n* (%)						
Antiplatelet therapy	27 (40%)	21 (32%)	0.34	2 (15%)	9 (33%)	10 (40%)
Oral anticoagulants	5 (8%)	1 (2%)	0.10	0 (0%)	1 (4%)	0 (0%)
Statins	23 (34%)	21 (32%)	0.81	2 (15%)	9 (33%)	10 (40%)
Clinical features, *n* (%)						
Aggressive behaviour	2 (3%)	3 (5%)	0.62	0 (0%)	2 (8%)	1 (4%)
Gait disturbance	16 (24%)	6 (9%)	0.024	1 (8%)	3 (11%)	2 (8%)
Recurrent falls	9 (13%)	3 (5%)	0.08	1 (8%)	1 (4%)	1 (4%)
TFNE	8 (12%)	1 (2%)	0.015	0 (0%)	1 (4%)	0 (0%)

Abbreviations: Aβ, amyloid beta; AD, Alzheimer's disease; AD‐MCI, mild cognitive impairment due to Alzheimer's disease; CAA, cerebral amyloid angiopathy; H, hemorrhagic marker; HC, healthy controls; IQR, Interquartile range; MCI, mild cognitive impairment; NH, non‐hemorrhagic marker; TFNE, transient focal neurological episode.

* The CAA 2.0 NH group (*n* = 65) comprised patients from the MCI‐NH (*n* = 13), AD‐NH (*n* = 27), and AD‐MCI‐NH (*n* = 25) groups, as shown in the adjacent columns.

^†^ Compared by Mann–Whitney *U* test or Pearson χ^2^ test.

### 3.2 CSF‐based neurochemical dementia diagnostics

**FIGURE 3:**
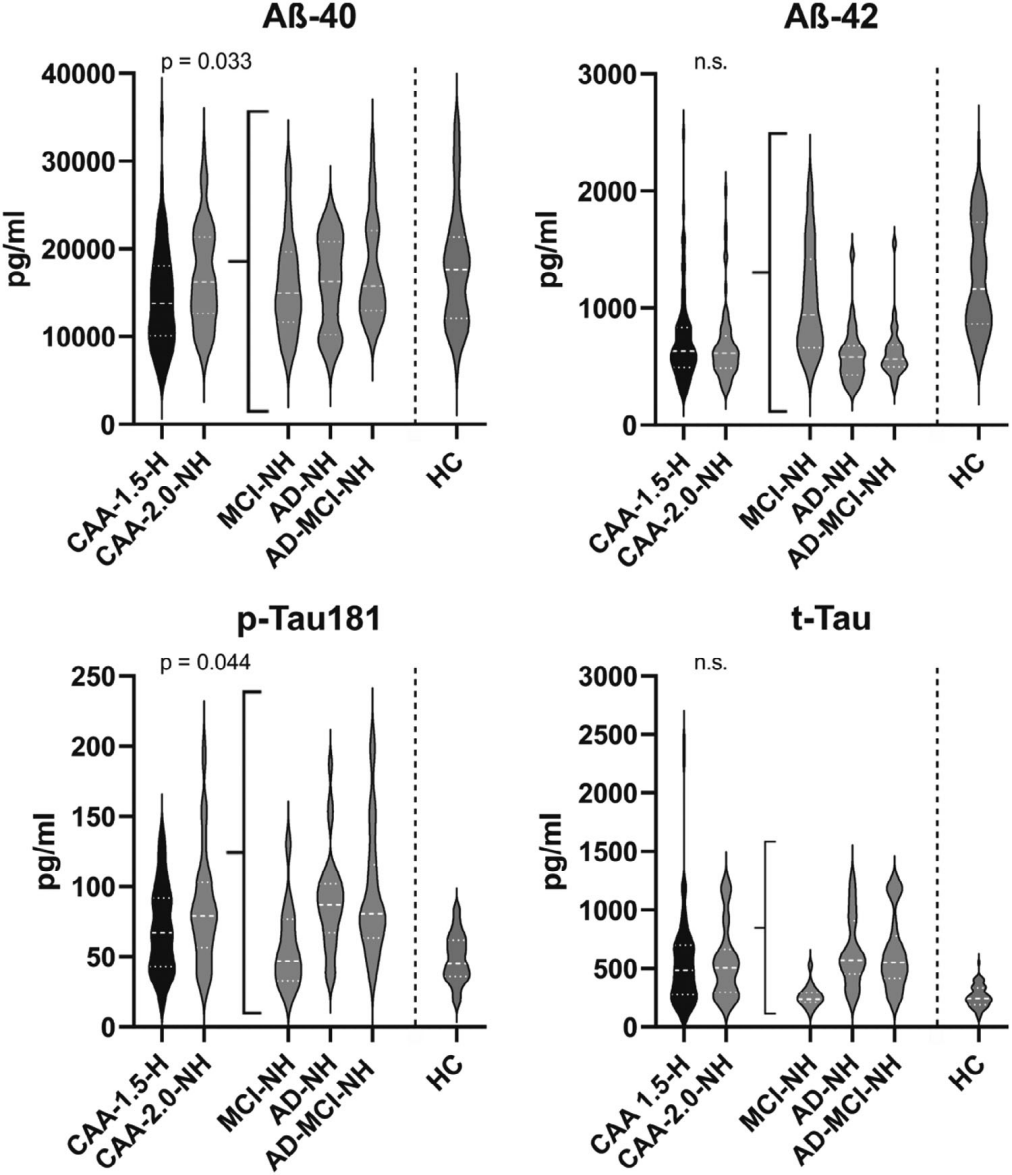
Violin plots of core cerebrospinal fluid biomarker levels. Graphical intergroup comparison of Aβ40, Aβ42, p‐tau181, and t‐tau levels using violin plots. Horizontal dashed lines within the violin plots indicate median values and interquartile ranges. The CAA 2.0 NH group (*n* = 65) includes patients from the MCI‐NH (*n* = 13), AD‐NH (*n* = 27), and AD‐MCI‐NH (*n* = 25) subgroups. Healthy controls are additionally displayed on the right side of the dashed separator line for visual comparison. Aβ, amyloid beta; AD, Alzheimer's disease; AD‐MCI, mild cognitive impairment due to Alzheimer's disease; CAA, cerebral amyloid angiopathy; H, hemorrhagic; HC, healthy controls; MCI, mild cognitive impairment; NH, non‐hemorrhagic; n.s., non‐significant; p‐tau, phosphorylated tau; t‐tau, total tau

Patients classified as CAA 2.0 NH showed higher Aß40 levels than those diagnosed with CAA 1.5 H (15,808 pg/ml [12,421–21,341] vs. 13,792 pg/ml [10,081–18,063], *p* = 0.033); see Figure 3 for violin plots. In addition, p‐tau181 concentrations were modestly higher in patients with CAA 2.0 NH (79 pg/ml [57–103] vs. 67 pg/ml [43–92], *p* = 0.044), whereas Aβ42 (617 pg/ml [478–761] vs. 634 pg/ml [492–834], *p* = 0.47) and t‐tau concentrations (507 pg/ml [297–663] vs. 468 pg/ml [275–698], *p* = 0.62) did not differ significantly between groups. In unadjusted analyses, p‐tau181 differences did not survive Bonferroni correction (*p* = 0.044). However, after adjustment for age, sex, and NDD biomarker assay type (see Table S7, Model 1, in supporting information) as well as after additional accounting for MRI field strength and hemosiderin‐sensitive sequence type (see Table S7, Model 2), p‐tau181 was significantly higher in CAA 2.0 NH (Model 1: *p* = 0.010; Model 2: *p* = 0.014), likely because adjustment for confounders unmasked the underlying association, consistent with the high proportion of concomitant AD pathology in this group (52/65, 80%). Findings for Aβ40, Aβ42, and t‐tau remained materially unchanged after adjustment.

Compared to the other etiologies of cognitive impairment, patients with CAA 2.0 NH had CSF profiles comparable to those of their original diagnostic category. The only exception was slightly lower Aß42 levels in patients with versus without non‐hemorrhagic imaging markers and AD‐MCI, which did not remain significant in adjusted analyses after Bonferroni correction (see Tables S3–S5).

Compared to HC, patients with CAA 2.0 NH differed in levels of Aß42 (HC: 1165 pg/ml [864–1733] vs. CAA 2.0 NH: 617 pg/ml [487–761], *p* < 0.001), p‐tau181 (45 pg/ml (36–62) vs. 79 pg/ml [57–103], *p* < 0.001), and t‐tau (245 pg/ml [188–334] vs. 507 pg/ml [297–663], *p* < 0.001). However, the levels of Aß40 were not different between the two groups (17,649 pg/ml [12,085–21,340] vs. 15,808 pg/ml [12,421–21,341], *p* = 0.35); see Table S6. CSF biomarker levels were additionally compared according to diagnostic certainty, both in the overall cohort fulfilling Boston Criteria 2.0 and separately in patients fulfilling hemorrhagic CAA criteria according to Boston Criteria 1.5, stratified by possible versus probable CAA. No significant differences in Aβ‐40, Aβ42, p‐tau181, or t‐tau levels were observed between groups, including after adjustment for potential confounders and multiple comparisons, suggesting no substantial association with diagnostic certainty level in the present cohort; see Tables S8A and S8B in supporting information.

Exploratory subgroup analyses stratified by AD CSF biomarker status revealed that CAA 2.0 NH patients with a concomitant AD CSF profile showed lower Aβ40 concentrations compared to those without an AD CSF profile (14,033 pg/ml vs. 17,318 pg/ml, *p* < 0.001), although this difference showed only a borderline statistical trend after adjustment for confounders (*p* = 0.05; Table S9A in supporting information). Notably, Aβ40 concentrations in CAA 2.0 NH patients with an AD CSF profile were comparable to those observed in CAA 1.5 H (14,033 pg/ml vs. 13,792 pg/ml, *p* = 0.65; Table S9B). Likewise, Aβ40 concentrations did not differ between CAA 1.5 H and CAA 2.0 NH among patients with an AD CSF profile, whereas higher Aβ40 concentrations persisted in CAA 2.0 NH among patients without an AD CSF profile after adjustment for potential confounders (Tables S9C and S9D).

## 4 DISCUSSION

Previous studies reported characteristic CSF biomarker alterations in patients with CAA diagnosed based on hemorrhagic imaging markers, particularly reduced Aβ40 concentrations. Patients reclassified as CAA 2.0 NH based on non‐hemorrhagic markers in our memory clinic cohort did not exhibit the reduction in Aβ40 concentrations previously observed in hemorrhagic CAA. Moreover, Aβ40 concentrations did not differ substantially between patients with CAA 2.0 NH and patients with AD, AD‐MCI, MCI, or HCs. Several explanations may account for these findings, including limited specificity of non‐hemorrhagic imaging markers in memory‐clinic cohorts, stage‐dependent differences in CAA manifestation, and confounding effects of concomitant neurodegenerative pathology on CSF biomarker interpretation.

Non‐hemorrhagic imaging markers were detected in 107/358 (29.9%) patients, resulting in 65 additional patients fulfilling possible CAA criteria according to Boston Criteria 2.0. The modification of Boston Criteria 2.0 allowing diagnosis of possible CAA based exclusively on non‐hemorrhagic MRI markers has the potential to result in overdiagnosis of CAA, particularly in memory clinic populations in whom such MRI changes are common due to aging and other small vessel pathologies.^3^, ^6^, ^12^, ^13^ This is consistent with findings from a previous memory clinic study reporting an even higher prevalence of non‐hemorrhagic MRI markers and greater number of patients reclassified as possible CAA.^7^ As intended by its developers, incorporation of non‐hemorrhagic markers serves the dual purpose of increasing diagnostic sensitivity while aiming to maintain specificity and identify CAA at earlier disease stages.^3^ However, the higher number of patients fulfilling possible CAA criteria raises the question whether these imaging markers are sufficiently specific for CAA in memory clinic populations or may additionally reflect aging, arteriosclerotic small vessel disease, or concomitant AD.^7^ The expanded diagnostic criteria may therefore also complicate interpretation of CSF biomarkers by increasing biological heterogeneity within the CAA 2.0 NH group.^14^ Because all patients with isolated non‐hemorrhagic markers can, by definition, only reach the “possible” CAA category, associated with a lower likelihood of underlying CAA pathology compared to “probable” CAA and likely enriched for mixed vascular and AD‐related pathology, these findings underscore the need for complementary biomarker approaches—such as CSF profiling—to improve diagnostic specificity beyond imaging‐based criteria alone. Consequently, the absence of characteristic CAA‐associated CSF alterations may in part reflect enrichment for mixed vascular or concomitant AD‐related pathology in memory clinic populations. Nevertheless, additional analyses stratified according to diagnostic certainty (possible vs. probable CAA according to Boston Criteria 2.0) yielded comparable findings, suggesting that the observed results were not solely driven by differences in diagnostic classification. A recent study involving 45 cognitively impaired or asymptomatic individuals without hemorrhagic markers also reported limited diagnostic accuracy of Boston Criteria 2.0 for histopathologically confirmed CAA (sensitivity 28.6%, specificity 65.3%).^15^ Because the Boston Criteria were initially developed to identify spontaneous ICH etiology, they may be more strongly calibrated toward identifying advanced CAA pathology associated with micro‐ and macrohemorrhages.^16^ Consequently, one plausible explanation for absence of Aβ40 reduction in patients classified by non‐hemorrhagic markers might be the inclusion of individuals without underlying CAA pathology. If so, this finding would further highlight the value of CSF biomarker profiles in identifying true‐positive CAA cases.

Alternatively, the observed CSF profiles may reflect lesion type–specific differences or represent earlier, pre‐hemorrhagic stages of CAA. This aligns with the proposed pathophysiological models that describe a stepwise progression from non‐hemorrhagic to hemorrhagic CAA manifestations.^4^ Although patients with CAA 2.0 NH exhibited a lower prevalence of certain clinical features such as TFNE and gait disturbances compared to CAA 1.5 H, these differences should be interpreted cautiously, as TFNEs are closely linked to hemorrhagic lesions such as cortical superficial siderosis and convexity subarachnoid hemorrhage and are therefore expected to be less frequent in a non‐hemorrhagic cohort by definition. Similar age distributions between groups would further suggest that factors beyond chronological aging, such as genetic susceptibility, amyloid clearance capacity, or coexisting pathology, influence whether CAA manifests predominantly with hemorrhagic or non‐hemorrhagic lesions.^7^, ^17^, ^18^ However, because CSF Aβ alterations typically precede clinical and radiological findings by years in AD, and likely also in CAA, this explanation appears less probable.^19^, ^20^, ^21^ In this context, the potential influence of apolipoprotein E (*APOE*) genotype would have been of particular interest, as *APOE* ε4 and ε2 alleles are known to differentially affect amyloid deposition patterns and hemorrhagic manifestations of CAA.

A third factor to consider is the frequent comorbidity of CAA and AD, especially in cognitively impaired populations, with complex interactions at multiple levels. Impaired clearance of Aβ from the interstitial fluid via perivascular drainage pathways may result in deposition either as neuritic plaques within the brain parenchyma or as amyloid angiopathy along vessel walls.^22^ Vascular dysfunction in CAA may further exacerbate amyloid accumulation and clearance deficits observed in AD.^22^, ^23^, ^24^ In addition, inflammatory processes may increase Aβ42 retention, as CAA‐related inflammation has previously been associated with lower CSF Aβ42 concentrations compared to non‐inflammatory CAA.^8^ These overlapping mechanisms likely complicate interpretation of CSF biomarkers, particularly in cases of co‐appearance, thereby reducing their diagnostic utility. In our cohort, both probable AD and AD‐MCI cases with additional non‐hemorrhagic CAA markers retained CSF profiles dominated by AD pathology. Notably, even patients with MCI without underlying AD reclassified as possible CAA showed no Aβ40 reduction, further questioning the specificity of these non‐hemorrhagic‐markers for CAA in this population. Exploratory subgroup analyses stratified by AD‐CSF biomarker status revealed that CAA 2.0 NH patients with a concomitant AD CSF profile showed Aβ40 concentrations comparable to those observed in hemorrhagic CAA (CAA 1.5 H), whereas patients without an AD CSF profile showed Aβ40 levels indistinguishable from HCs. Whether the lower Aβ40 levels in the AD CSF profile subgroup reflect true comorbid vascular amyloid deposition, which is more prevalent in patients with AD pathology, or generalized amyloid‐clearance dysfunction in advanced AD cannot be determined without pathological verification. These stratified analyses should be interpreted with caution given the limited sample sizes, which may have been insufficient to detect moderate effect sizes. Clinically, correct identification of CAA is highly relevant when treating AD patients with anti‐Aβ monoclonal antibodies, given that coexisting CAA is recognized as a major risk factor for developing amyloid‐related imaging abnormalities (ARIAs).^25^ While the contribution of hemorrhagic markers to ARIA risk is established, little is known about the role of non‐hemorrhagic CAA markers.^7^, ^25^ The absence of a specific CSF profile in patients with non‐hemorrhagic CAA markers, coupled with their high prevalence among memory clinic patients, further complicates risk stratification for ARIAs associated with CAA.

The study's limitations are attributable to its observational and retrospective nature in a monocentric setting. Possibility of selection bias exists, as the study's participants were selected based on presence of CSF biomarkers and MRI scans with blood‐sensitive sequences alongside cognitive complaints. Therefore, we may have missed patients with CAA admitted for other reasons, such as TFNE and ischemic or hemorrhagic stroke, limiting generalizability. This principle also pertains to CAA diagnosis, which was solely based on MRI data without any pathologic confirmation. Nevertheless, our methodology reflects a realistic real‐world diagnostic scenario in settings in which cognitive decline is the primary concern. In addition, *APOE* genotype data were not systematically available in this retrospective cohort and could therefore not be included in the analyses, although *APOE* genotype is known to influence amyloid deposition patterns, hemorrhagic manifestations of CAA, and concomitant AD pathology.^26^ Although our sample represents one of the largest CAA CSF biomarker analyses to date, statistical power may still have been insufficient to detect subtle group differences, particularly in the exploratory subgroup analyses stratified by AD CSF profile. Furthermore, analyses of individual non‐hemorrhagic markers, such as severity of perivascular spaces or white matter hyperintensity patterns, as well as associations between CSF biomarkers and specific cognitive profiles, may provide additional mechanistic insights beyond the current imaging‐based classification approach but were beyond the scope of the present study. These analyses represent important directions for future research. Prospective and pathology‐verified studies in multicenter designs are needed to confirm our results.

In conclusion, non‐hemorrhagic MRI markers alone did not correspond to distinct CSF alterations characteristic of CAA, thereby questioning their validity in memory clinic populations or suggesting lesion type–dependent CSF variability. These results underscore the importance of integrated multimodal diagnostic approaches and future prospective studies to clarify the clinical significance of non‐hemorrhagic CAA markers in cognitively impaired patients. Stratification by concomitant AD pathology may help identify biologically distinct subgroups within non‐hemorrhagic CAA populations and warrants further investigation in pathology‐verified cohorts.

## CONFLICT OF INTEREST STATEMENT

J.A.S. reports receiving speaker honoraria from Eisai and Eli Lilly and Company, unrelated to the submitted work. P.L. reports consulting fees from the Luxembourg Institute of Health, Mikrogen GmbH, the European Commission, Roboscreen GmbH, and Fujirebio Europe, and speaker honoraria from Instand e.V. and Biofarm Poland. T.J.O. reports institutional grants/contracts from Siemens Healthineers and Roche; speaker honoraria from Siemens Healthineers and Lilly; payments for expert testimony from Siemens Healthineers and Lilly, and institutional payments from Biogen; travel support from Siemens Healthineers; and membership of the German Dementia Registry (DEMREG) Steering Committee. All other authors declare that they have no competing interests. Author disclosures are available in the Supporting Information.

## ETHICS APPROVAL AND CONSENT TO PARTICIPATE

The ethics committee of the University Erlangen‐Nuremberg approved the study protocol (Nr. 20_21 Bc) and centrally granted a waiver of informed consent.

## Supporting information



Supporting Information

Supporting Information
