# Supplementary material for: Cerebrospinal fluid biomarkers for cerebral amyloid angiopathy with non‐hemorrhagic imaging marker
Source: Alzheimers Dement (Amst). 2026 Aug 19;18(3):e70431. doi: 10.1002/dad2.70431 (PMC13490837; doi:10.1002/dad2.70431)
Supplement: Supplementary file 2 [file DAD2-18-e70431-s002.docx]

**Supplementary Material**

*Sekita et al.	Cerebrospinal fluid biomarkers for cerebral amyloid angiopathy with non-hemorrhagic imaging marker*

**Content:**

**sTable 1:	MRI characteristics across diagnostic groups.**

**sTable 2:	Intergroup comparison of patient characteristics between CAA-2.0-NH and alternative etiologies of cognitive impairment**

**sTable 3:	Intergroup comparison of patient characteristics and core cerebrospinal fluid biomarker concentrations between reclassified former MCI patients with NH and MCI patients without NH.**

**sTable 4:	Intergroup comparison of patient characteristics and core cerebrospinal fluid biomarker concentrations between reclassified former AD patients with NH and AD patients without NH.**

**sTable 5:	Intergroup comparison of patient characteristics and core cerebrospinal fluid biomarker concentrations between reclassified former AD-MCI patients with NH and AD-MCI patients without NH.**

**sTable 6:	Intergroup comparison of patient characteristics and core cerebrospinal fluid biomarker concentrations between CAA-2.0-NH and HC.**

**sTable 7:	Intergroup comparison of core cerebrospinal fluid biomarker levels between patients with CAA-2.0-NH and CAA-1.5-H adjusted for potential confounders.**

**sTable 8:	Comparison of CSF biomarker concentrations stratified by diagnostic certainty evaluated by Boston Criteria version 2.0 in patients (A) with hemorrhagic and/or non-hemorrhagic CAA marker and (B) with mandatory presence of hemorrhagic CAA marker**

**sTable 9:	Comparison of CSF biomarker concentrations according to AD CSF biomarker profile in patients with CAA-2.0-NH and CAA-1.5-H**

**Supplementary Table 1: MRI characteristics across diagnostic groups.**

| **Parameter** | **CAA-1.5-H (n=67)** | **CAA-2.0-NH (n=65)** | **MCI & AD & AD-MCI without NH (n=162)** | **HC (n=64)** | **p-value*** |
| --- | --- | --- | --- | --- | --- |
| MRI field strength 1.5T, n (%) | 46 (69%) | 42 (65%) | 115 (71%) | 41 (64%) | 0.69 |
| MRI field strength 3T, n (%) | 21 (31%) | 23 (35%) | 47 (29%) | 23 (36%) |  |
| GRE sequence, n (%) | 50 (75%) | 54 (83%) | 136 (84%) | 52 (81%) | 0.41 |
| SWI sequence, n (%) | 17 (25%) | 11 (17%) | 26 (16%) | 12 (19%) |  |
| Number of lobar microbleeds, median (IQR) | 2 (1-3) | 0 (0-0) | 0 (0-0) | 0 (0-0) | <0.001 |
| White matter hyperintensities, n (%) | 39 (58%) | 35 (54%) | 0 (0%) | 9 (14%) | <0.001 |
| Severe perivascular spaces, n (%) | 23 (34%) | 39 (60%) | 0 (0%) | 6 (9%) | <0.001 |

*Compared by Mann–Whitney U-test or Pearson’s χ2-test.

Abbreviations: AD, Alzheimer’s disease; AD-MCI, mild cognitive impairment due to Alzheimer’s disease; CAA, cerebral amyloid angiopathy; GRE, gradient-echo; H, hemorrhagic marker; HC, healthy controls; IQR, interquartile range; MCI, mild cognitive impairment; NH, non-hemorrhagic marker; SWI, susceptibility-weighted imaging.

**Supplementary Table 2: Intergroup comparison of patient characteristics between CAA-2.0-NH and alternative etiologies of cognitive impairment**

| **Characteristics** | **MCI & AD & AD-MCI without NH (n=162)** | **CAA-2.0-NH**  **(n=65)** | **p-value*** |
| --- | --- | --- | --- |
| Age, years, median (IQR) | 69 (59-75) | 73 (67-77) | 0.002 |
| Sex, female, n (%) | 72 (44%) | 37 (57%) | 0.09 |
| Prior medical history |  |  |  |
| Intracerebral hemorrhage, n (%) | 3 (2%) | 0 (0%) | 0.27 |
| Ischemic stroke, n (%) | 14 (9%) | 10 (15%) | 0.14 |
| Transient ischemic attack, n (%) | 5 (3%) | 2 (3%) | 1.00 |
| Subarachnoid hemorrhage, n (%) | 2 (1%) | 0 (0%) | 0.37 |
| Arterial hypertension, n (%) | 108 (67%) | 44 (68%) | 0.88 |
| Diabetes mellitus, n (%) | 20 (12%) | 7 (11%) | 0.74 |
| Prior medication |  |  |  |
| Antiplatelet therapy, n (%) | 53 (33%) | 21 (32%) | 0.95 |
| Oral anticoagulants, n (%) | 11 (7%) | 1 (2%) | 0.11 |
| Statins, n (%) | 52 (32%) | 21 (32%) | 0.98 |
| Clinical features |  |  |  |
| Aggressive behaviour, n (%) | 4 (3%) | 3 (5%) | 0.40 |
| Gait disturbance, n (%) | 10 (6%) | 6 (9%) | 0.42 |
| Recurrent falls, n (%) | 11 (7%) | 3 (5%) | 0.54 |
| TFNE, n (%) | 5 (3%) | 1 (2%) | 0.51 |

*Compared by Mann–Whitney U-test or Pearson’s χ2-test.

Abbreviations: Aβ, β-amyloid; AD; Alzheimer´s disease; AD-MCI, mild cognitive impairment due to Alzheimer’s disease; CAA, Cerebral amyloid angiopathy; IQR, Interquartile range; MCI, mild cognitive impairment; NH, non-hemorrhagic marker; TFNE, transient focal neurological episode.

**Supplementary Table 3: Intergroup comparison of patient characteristics and core cerebrospinal fluid biomarker concentrations between reclassified former MCI patients with NH and MCI patients without NH.**

| **Characteristics** | **MCI without NH**  **(n=63)** | **MCI with NH**  **(n=13)** | **p-value*** | **Regression coefficient B** | **ß** | **p-value**** |
| --- | --- | --- | --- | --- | --- | --- |
| Age, years, median (IQR) | 67 (56-74) | 69 (61-75) | 0.37 |  | | |
| Sex, female, n (%) | 26 (42%) | 7 (54%) | 0.41 |  |  |  |
| Prior medical history, n (%) |  |  |  |  |  |  |
| Intracerebral hemorrhage | 2 (3%) | 0 (0%) | 0.52 |  |  |  |
| Ischemic stroke | 10 (16%) | 4 (31%) | 0.21 |  |  |  |
| Transient ischemic attack | 2 (3%) | 0 (0%) | 0.52 |  |  |  |
| Subarachnoid hemorrhage | 2 (3%) | 0 (0%) | 0.52 |  |  |  |
| Arterial hypertension | 46 (73%) | 7 (54%) | 0.17 |  |  |  |
| Diabetes mellitus | 9 (14%) | 0 (0%) | 0.15 |  |  |  |
| Prior medication, n (%) |  |  |  |  |  |  |
| Antiplatelet therapy | 21 (33%) | 2 (15%) | 0.20 |  |  |  |
| Oral anticoagulants | 5 (8%) | 0 (0%) | 0.29 |  |  |  |
| Statins | 20 (32%) | 2 (15%) | 0.24 |  |  |  |
| Clinical features, n (%) |  |  |  |  |  |  |
| Aggressive behaviour | 0 (0%) | 0 (0%) | - |  |  |  |
| Gait disturbance | 7 (11%) | 1 (8%) | 0.72 |  |  |  |
| Recurrent falls | 7 (11%) | 1 (8%) | 0.72 |  |  |  |
| TFNE | 2 (3%) | 0 (0%) | 0.52 |  |  |  |
| CSF biomarker concentrations, pg/ml; median (IQR) |  |  |  |  |  |  |
| Aß-40 | 16,291 (11,893 -20 527) | 14,997 (10,735  -19, 977) | 0.53 | -735.71 | -0.05 | 0.67 |
| Aß-42 | 1,175 (898  -1,488) | 817 (644-1,509) | 0.18 | -89.03 | -0.08 | 0.44 |
| p-Tau181 | 47 (36-60) | 47 (32-77) | 0.62 | 2.59 | 0.03 | 0.77 |
| t-Tau | 268 (197-335) | 232 (200-306) | 0.50 | -62.06 | -0.13 | 0.28 |

*Compared by Mann–Whitney U-test or Pearson’s χ2-test

**Adjusted p-values derived from multivariable linear regression analyses of primary (Aß-40) and secondary outcomes (Aß-42, p-Tau181, t-Tau) adjusted for age, sex, and assay type.

Abbreviations: Aβ, β-amyloid; AD; Cerebral amyloid angiopathy; CSF, cerebrospinal fluid; IQR, Interquartile range; MCI, mild cognitive impairment; NH, non-hemorrhagic marker; TFNE, transient focal neurological episode.

**Supplementary Table 4: Intergroup comparison of patient characteristics and core cerebrospinal fluid biomarker concentrations between reclassified former AD patients with NH and AD patients without NH.**

| **Characteristics** | **AD without NH**  **(n=49)** | **AD with NH**  **(n=27)** | **p-value*** | **Regression coefficient B** | **ß** | **p-value**** |
| --- | --- | --- | --- | --- | --- | --- |
| Age, years, median (IQR) | 70 (59-77) | 75 (69-77) | 0.035 |  | | |
| Sex, female, n (%) | 24 (49%) | 15 (56%) | 0.58 |  |  |  |
| Prior medical history, n (%) |  |  |  |  |  |  |
| Intracerebral hemorrhage | 0 (0%) | 0 (0%) | - |  |  |  |
| Ischemic stroke | 4 (8%) | 5 (19%) | 0.18 |  |  |  |
| Transient ischemic attack | 3 (6%) | 2 (7%) | 0.83 |  |  |  |
| Subarachnoid hemorrhage | 0 (0%) | 0 (0%) | - |  |  |  |
| Arterial hypertension | 29 (59%) | 22 (82%) | 0.048 |  |  |  |
| Diabetes mellitus | 5 (10%) | 3 (11%) | 0.90 |  |  |  |
| Prior medication, n (%) |  |  |  |  |  |  |
| Antiplatelet therapy | 13 (27%) | 9 (33%) | 0.53 |  |  |  |
| Oral anticoagulants | 5 (10%) | 1 (4%) | 0.32 |  |  |  |
| Statins | 14 (29%) | 9 (33%) | 0.67 |  |  |  |
| Clinical features, n (%) |  |  |  |  |  |  |
| Aggressive behaviour | 3 (6%) | 2 (8%) | 0.83 |  |  |  |
| Gait disturbance | 2 (4%) | 3 (11%) | 0.24 |  |  |  |
| Recurrent falls | 2 (4%) | 1 (4%) | 0.94 |  |  |  |
| TFNE | 3 (6%) | 1 (4%) | 0.65 |  |  |  |
| CSF biomarker concentrations, pg/ml; median (IQR) |  |  |  |  |  |  |
| Aß-40 | 16,118 (12,569  -20,948) | 16,301 (10,221  -20,851) | 0.85 | -310.89 | -0.03 | 0.79 |
| Aß-42 | 588 (499-709) | 581 (428-676) | 0.33 | -37.91 | -0.08 | 0.50 |
| p-Tau181 | 93 (71-122) | 87 (67-102) | 0.38 | -6.14 | -0.08 | 0.52 |
| t-Tau | 622 (462-1,200) | 572 (452-908) | 0.14 | -137.32 | -0.18 | 0.13 |

*Compared by Mann–Whitney U-test or Pearson’s χ2-test.

**Adjusted p-values derived from multivariable linear regression analyses of primary (Aß-40) and secondary outcomes (Aß-42, p-Tau181, t-Tau) adjusted for age, sex, and assay type.

Abbreviations: Aβ, β-amyloid; AD; Alzheimer´s disease; CAA, Cerebral amyloid angiopathy; CSF, cerebrospinal fluid; IQR, Interquartile range; NH, non-hemorrhagic marker; TFNE, transient focal neurological episode.

**Supplementary Table 5:** **Intergroup comparison of patient characteristics and core cerebrospinal fluid biomarker concentrations between reclassified former AD-MCI patients with NH and AD-MCI patients without NH.**

| **Characteristics** | **AD-MCI without NH (n=50)** | **AD-MCI with NH**  **(n=25)** | **p-value*** | **Regression coefficient B** | **ß** | **p-value** |
| --- | --- | --- | --- | --- | --- | --- |
| Age, years, median (IQR) | 71 (63-75) | 73 (69-75) | 0.19 |  | | |
| Sex, female, n (%) | 22 (44%) | 15 (60%) | 0.19 |  |  |  |
| Prior medical history, n (%) |  |  |  |  |  |  |
| Intracerebral hemorrhage | 1 (2%) | 0 (0%) | 0.48 |  |  |  |
| Ischemic stroke | 0 (0%) | 1 (4%) | 0.16 |  |  |  |
| Transient ischemic attack | 0 (0%) | 0 (0%) | - |  |  |  |
| Subarachnoid hemorrhage | 0 (0%) | 0 (0%) | - |  |  |  |
| Arterial hypertension | 33 (66%) | 15 (60%) | 0.61 |  |  |  |
| Diabetes mellitus | 6 (12%) | 4 (16%) | 0.63 |  |  |  |
| Prior medication, n (%) |  |  |  |  |  |  |
| Antiplatelet therapy | 19 (38%) | 10 (40%) | 0.87 |  |  |  |
| Oral anticoagulants | 1 (2%) | 0 (0%) | 0.48 |  |  |  |
| Statins | 18 (36%) | 10 (40%) | 0.74 |  |  |  |
| Clinical features, n (%) |  |  |  |  |  |  |
| Aggressive behaviour | 1 (2%) | 1 (4%) | 0.61 |  |  |  |
| Gait disturbance | 1 (2%) | 2 (8%) | 0.21 |  |  |  |
| Recurrent falls | 2 (4%) | 1 (4%) | 1.00 |  |  |  |
| TFNE | 0 (0%) | 0 (0%) | - |  |  |  |
| CSF biomarker concentrations, pg/ml; median (IQR) |  |  |  |  |  |  |
| Aß-40 | 16,434 (13,626  -21,823) | 15,641(12,945 -22,219) | 0.57 | -948.58 | -0.07 | 0.52 |
| Aß-42 | 639 (554-807) | 554 (490-683) | 0.012^#^ | -147.85 | -0.27 | 0.026 |
| p-Tau181 | 84 (62-110) | 80 (63-119) | 0.91 | -5.50 | -0.06 | 0.64 |
| t-Tau | 533 (400-760) | 517 (404-812) | 0.94 | -22.13 | -0.03 | 0.80 |

*Compared by Mann–Whitney U-test or Pearson’s χ2-test.

**Adjusted p-values derived from multivariable linear regression analyses of primary (Aß-40) and secondary outcomes (Aß-42, p-Tau181, t-Tau) adjusted for age, sex, and assay type.

^#^ Significant after adjustment using Bonferroni correction (adjusted significance threshold for secondary outcomes: α = 0.017).

Abbreviations: Aβ, β-amyloid; CAA, Cerebral amyloid angiopathy; CSF, cerebrospinal fluid; IQR, Interquartile range; AD-MCI, mild cognitive impairment due to Alzheimer’s disease; NH, non-hemorrhagic marker; TFNE, transient focal neurological episode.

**Supplementary Table 6:** **Intergroup comparison of patient characteristics and core cerebrospinal fluid biomarker concentrations between CAA-2.0-NH and HC.**

| **Characteristics** | **HC**  **(n=64)** | **CAA 2.0-NH**  **(n=65)** | **p-value*** | **Regression coefficient B** | **ß** | **p-value**** |
| --- | --- | --- | --- | --- | --- | --- |
| Age, years, median (IQR) | 58 (53-64) | 73 (67-77) | <0.001 |  | | |
| Sex, female, n (%) | 23 (36%) | 37 (57%) | 0.017 |  |  |  |
| Prior medical history, n (%) |  |  |  |  |  |  |
| Intracerebral hemorrhage | 0 (0%) | 0 (0%) | - |  |  |  |
| Ischemic stroke | 0 (0%) | 10 (15%) | 0.001 |  |  |  |
| Transient ischemic attack | 0 (0%) | 2 (3%) | 0.16 |  |  |  |
| Subarachnoid hemorrhage | 0 (0%) | 0 (0%) | - |  |  |  |
| Arterial hypertension | 39 (61%) | 44 (68%) | 0.42 |  |  |  |
| Diabetes mellitus | 8 (13%) | 7 (11%) | 0.76 |  |  |  |
| Prior medication, n (%) |  |  |  |  |  |  |
| Antiplatelet therapy | 10 (16%) | 21 (32%) | 0.027 |  |  |  |
| Oral anticoagulants | 1 (1.3%) | 1 (2%) | 0.99 |  |  |  |
| Statins | 10 (16%) | 21 (32%) | 0.027 |  |  |  |
| Clinical features, n (%) |  |  |  |  |  |  |
| Aggressive behaviour | 0 (0%) | 3 (5%) | 0.08 |  |  |  |
| Gait disturbance | 0 (0%) | 6 (9%) | 0.013 |  |  |  |
| Recurrent falls | 0 (0%) | 3 (5%) | 0.082 |  |  |  |
| TFNE | 0 (0%) | 1 (2%) | 0.32 |  |  |  |
| CSF biomarker concentrations, pg/ml; median (IQR) |  |  |  |  |  |  |
| Aß-40 | 17,649 (12,085  -21,340) | 15,808 (12,421  -21,341) | 0.35 | -1,641.92 | -0.13 | 0.20 |
| Aß-42 | 1,165 (864  -1,733) | 617 (487-761) | <0.001^#^ | -488.52 | -0.49 | <0.001^#^ |
| p-Tau181 | 45 (36-62) | 79 (57-103) | <0.001^#^ | 25.48 | 0.36 | <0.001^#^ |
| t-Tau | 245 (188-334) | 507 (297-663) | <0.001^#^ | 230.22 | 0.42 | <0.001^#^ |

*Compared by Mann–Whitney U-test or Pearson’s χ2-test.

**Adjusted p-values derived from multivariable linear regression analyses of primary (Aß-40) and secondary outcomes (Aß-42, p-Tau181, t-Tau) adjusted for age, sex, and assay type.

^#^ Significant after adjustment using Bonferroni correction (adjusted significance threshold for secondary outcomes: α = 0.017).

Abbreviations: Aβ, β-amyloid; CAA, Cerebral amyloid angiopathy; CSF, cerebrospinal fluid; HC, healthy controls; IQR, Interquartile range; NH, non-hemorrhagic marker; TFNE, transient focal neurological episode.

**Supplementary Table 7:** **Intergroup comparison of core cerebrospinal fluid biomarker levels between patients with CAA-2.0-NH and CAA-1.5-H adjusted for potential confounders.**

| CSF biomarker concentrations, pg/ml; median (IQR) | Model 1: Adjusted for age, sex, assay type | | | Model 2: Adjusted for age, sex, assay type, MRI field strength, hemosiderin-sensitive MRI sequence type | | |
| --- | --- | --- | --- | --- | --- | --- |
|  | Regression coefficient B | β | p-value | Regression coefficient B | β | p-value |
| Aβ-40 | 2359.19 | 0.20 | 0.020 | 2309.35 | 0.20 | 0.017 |
| Aβ-42 | -45.13 | -0.06 | 0.48 | -53.33 | -0.073 | 0.40 |
| p-Tau181 | 16.27 | 0.22 | 0.010^#^ | 15.75 | 0.22 | 0.014^#^ |
| t-Tau | 16.75 | 0.02 | 0.80 | 22.49 | 0.03 | 0.73 |

Adjusted p-values derived from multivariable linear regression analyses of primary (Aß-40) and secondary outcomes (Aß-42, p-Tau181, t-Tau). Model 1 was adjusted for age, sex, and assay type. Model 2 was additionally adjusted for MRI field strength (1.5T vs. 3T) and hemosiderin-sensitive MRI sequence type (SWI vs. GRE). Regression coefficients (B), standardized beta coefficients (β) and p-values are shown.

^#^ Significant after adjustment using Bonferroni correction (adjusted significance threshold for secondary outcomes: α = 0.017).

Abbreviations: Aβ, β-amyloid; CAA, cerebral amyloid angiopathy; CSF, cerebrospinal fluid; GRE, gradient-echo; H, hemorrhagic; IQR, interquartile range; NH, non-hemorrhagic marker; SWI, susceptibility-weighted imaging.

**Supplementary Table 8: Comparison of CSF biomarker concentrations stratified by diagnostic certainty evaluated by Boston Criteria version 2.0 in patients (A) with hemorrhagic and/or non-hemorrhagic CAA marker and (B) with mandatory presence of hemorrhagic CAA marker**

| **A** | | | | | | |
| --- | --- | --- | --- | --- | --- | --- |
| CSF biomarker concentrations,  pg/ml; median (IQR) | **Possible CAA**  **(n=76)** | **Probable CAA**  **(n=56)** | **p-value*** | **Regression coefficient B** | **ß** | **p-value**** |
| Aß-40 | 14,997 (11,567  -20,196) | 14,552 (9,901  -19,136) | 0.34 | -958.05 | -0.08 | 0.33 |
| Aß-42 | 617 (493-814) | 629 (487-865) | 0.62 | 54.95 | 0.08 | 0.39 |
| p-Tau181 | 77 (53-102) | 69 (42-92) | 0.25 | -10.67 | -0.14 | 0.09 |
| t-Tau | 507 (291-713) | 489 (275-685) | 0.57 | -96.33 | -0.13 | 0.14 |
| **B** |  |  |  |  |  |  |
| CSF biomarker concentrations,  pg/ml; median (IQR) | **Possible CAA (n=11)** | **Probable CAA (n=56)** | **p-value*** | **Regression coefficient B** | **ß** | **p-value**** |
| Aß-40 | 11,950 (10,062  -13,381) | 14,552 (9,901  -19,136) | 0.13 | 3,331.96 | 0.22 | 0.07 |
| Aß-42 | 737 (493-834) | 629 (487-865) | 0.88 | 51.59 | 0.05 | 0.70 |
| p-Tau181 | 53 (43-76) | 69 (42-92) | 0.34 | 10.85 | 0.13 | 0.27 |
| t-Tau | 443 (265-943) | 489 (275-685) | 0.72 | -278.98 | -0.25 | 0.035 ^ns^ |

*Compared by Mann–Whitney U-test or Pearson’s χ2-test.

**Adjusted p-values derived from multivariable linear regression analyses of primary (Aß-40) and secondary outcomes (Aß-42, p-Tau181, t-Tau) adjusted for age, sex, and assay type.

^ns^ Not significant after adjustment using Bonferroni correction (adjusted significance threshold for secondary outcomes: α = 0.017).

Abbreviations: Aβ, β-amyloid; CAA, Cerebral amyloid angiopathy; CSF, cerebrospinal fluid; IQR, Interquartile range.

**Supplementary Table 9: Comparison of CSF biomarker concentrations according to AD CSF biomarker profile in patients with CAA-2.0-NH and CAA-1.5-H**

| **A** | | | | | | |
| --- | --- | --- | --- | --- | --- | --- |
| CSF biomarker concentrations,  pg/ml; median (IQR) | **CAA-2.0-NH without AD CSF profile**  **(n=39)** | **CAA-2.0-NH with AD CSF profile (n=26)** | **p-value*** | **Regression coefficient B** | **ß** | **p-value**** |
| Aß-40 | 17,318 (12,927  -22,521) | 14,033 (11,186  -16,345) | <0.001 | -2,559.70 | -0.23 | 0.05 |
| Aß-42 | 710 (628-875) | 472 (378-527) | <0.001^#^ | -332.68 | -0.50 | <0.001^#^ |
| p-Tau181 | 77 (44-102) | 85 (70-106) | 0.60 | 19.40 | 0.24 | 0.07 |
| t-Tau | 369 (243-604) | 583 (477-917) | 0.14 | 193.72 | 0.31 | 0.016^#^ |
| **B** | | | | | | |
| CSF biomarker concentrations,  pg/ml; median (IQR) | **CAA-1.5-H**  **(n=67)** | **CAA-2.0-NH with AD CSF profile (n=26)** | **p-value*** | **Regression coefficient B** | **ß** | **p-value**** |
| Aß-40 | 13,792 (10,081-18,063) | 14,033 (11,186  -16,345) | 0.65 | 896.79 | 0.08 | 0.48 |
| Aß-42 | 634 (492-834) | 472 (378-527) | <0.001^#^ | -274.40 | -0.35 | 0.001^#^ |
| p-Tau181 | 67 (43-92) | 85 (70-106) | 0.002^#^ | 27.92 | 0.36 | <0.001^#^ |
| t-Tau | 468 (275-698) | 583 (477-917) | 0.031 | 134.21 | 0.16 | 0.15 |
|  |  |  |  |  |  |  |
| **C** |  |  |  |  |  |  |
| CSF biomarker concentrations,  pg/ml; median (IQR) | **CAA-1.5-H with AD CSF profile (n=18)** | **CAA-2.0-NH with AD CSF profile (n=26)** | **p-value*** | **Regression coefficient B** | **ß** | **p-value**** |
| Aß-40 | 14,298 (10,660-19,045) | 14,033 (11,186-16,345) | 0.99 | -369.08 | -0.04 | 0.83 |
| Aß-42 | 466 (386-558) | 472 (378-527) | 0.97 | -100.48 | -0.16 | 0.35 |
| p-Tau181 | 85 (68-96) | 85 (70-106) | 0.58 | 6.95 | 0.11 | 0.51 |
| t-Tau | 645 (475-795) | 583 (477-917) | 0.82 | -64.45 | -0.09 | 0.56 |
|  |  |  |  |  |  |  |
| **D** |  |  |  |  |  |  |
| CSF biomarker concentrations,  pg/ml; median (IQR) | **CAA-1.5-H without AD CSF profile**  **(n=49)** | **CAA-2.0-NH without AD CSF profile (n=39)** | **p-value*** | **Regression coefficient B** | **ß** | **p-value**** |
| Aß-40 | 13,381 (9,482-17,676) | 17,318 (12,927-22,521) | 0.07 | 3712.90 | 0.31 | 0.003^#^ |
| Aß-42 | 737 (606-887) | 710 (628-875) | 0.66 | 37.13 | 0.05 | 0.61 |
| p-Tau181 | 53 (38-89) | 77 (44-102) | 0.15 | 15.13 | 0.21 | 0.049 |
| t-Tau | 371 (246-685) | 369 (243-604) | 0.85 | 2.09 | 0.003 | 0.98 |

Comparison of CSF biomarker concentrations in patients with (A) CAA-2.0-NH with versus without an AD CSF biomarker profile; (B) CAA-2.0-NH with an AD CSF biomarker profile versus CAA-1.5-H; (C) CAA-2.0-NH versus CAA-1.5-H among patients with an AD CSF biomarker profile; and (D) CAA-2.0-NH versus CAA-1.5-H among patients without an AD CSF biomarker profile.

*Compared by Mann–Whitney U-test or Pearson’s χ2-test.

**Adjusted p-values derived from multivariable linear regression analyses of primary (Aß-40) and secondary outcomes (Aß-42, p-Tau181, t-Tau) adjusted for age, sex, and assay type.

^#^ Significant after adjustment using Bonferroni correction (adjusted significance threshold for secondary outcomes: α = 0.017).

Abbreviations: Aβ, β-amyloid; AD, Alzheimer's disease; CAA, cerebral amyloid angiopathy; CSF, cerebrospinal fluid; IQR, interquartile range; H, hemorrhagic marker; NH, non-hemorrhagic marker.
